# Highly Robust and Wearable Facial Expression Recognition via Deep-Learning-Assisted, Soft Epidermal Electronics

**DOI:** 10.34133/2021/9759601

**Published:** 2021-07-15

**Authors:** Meiqi Zhuang, Lang Yin, Youhua Wang, Yunzhao Bai, Jian Zhan, Chao Hou, Liting Yin, Zhangyu Xu, Xiaohui Tan, YongAn Huang

**Affiliations:** ^1^Information Engineering College, Capital Normal University, Beijing 100048, China; ^2^State Key Laboratory of Digital Manufacturing Equipment and Technology, Huazhong University of Science and Technology, Wuhan 430074, China; ^3^Flexible Electronics Research Center, Huazhong University of Science and Technology, Wuhan 430074, China

## Abstract

The facial expressions are a mirror of the elusive emotion hidden in the mind, and thus, capturing expressions is a crucial way of merging the inward world and virtual world. However, typical facial expression recognition (FER) systems are restricted by environments where faces must be clearly seen for computer vision, or rigid devices that are not suitable for the time-dynamic, curvilinear faces. Here, we present a robust, highly wearable FER system that is based on deep-learning-assisted, soft epidermal electronics. The epidermal electronics that can fully conform on faces enable high-fidelity biosignal acquisition without hindering spontaneous facial expressions, releasing the constraint of movement, space, and light. The deep learning method can significantly enhance the recognition accuracy of facial expression types and intensities based on a small sample. The proposed wearable FER system is superior for wide applicability and high accuracy. The FER system is suitable for the individual and shows essential robustness to different light, occlusion, and various face poses. It is totally different from but complementary to the computer vision technology that is merely suitable for simultaneous FER of multiple individuals in a specific place. This wearable FER system is successfully applied to human-avatar emotion interaction and verbal communication disambiguation in a real-life environment, enabling promising human-computer interaction applications.

## 1. Introduction

Facial expression is one of the main ways to convey emotional states and intentions, which contains rich emotional and cognitive information and is of practical importance in sociable robotics, medical treatment, driver fatigue surveillance, and especially human-computer interaction [1–3]. With the rapid development of artificial intelligence, the automatic facial expression recognition (FER) has attracted notable research interests via computer vision technologies [4, 5], which however fails to solve four main problems: illumination changes, occlusions, pose variations, and constrained positions. Besides, with the increasing privacy protection, the computer-vision-based FER becomes a major barrier in specific FER since it still depends on high-resolution images; even if a few attempts have been made [6, 7], the privacy security of computer vision remains controversial. Besides, computer-vision-based method is constrained to a fixed environment with the need for visual monitoring of the face which limited the applicability in a daily life environment. A GAN-based model was proposed to generate images with different expressions under arbitrary poses for multiview FER [8]. A novel region attention network was proposed to adaptively capture the importance of facial regions for occlusion and pose variant FER [9]. However, the problem of identity bias is commonly ignored. Moreover, generating diverse data accounts for additional time consumption, and the combination of these multiple data leads to high dimension which significantly decreases the computational efficiency of the network. 3D FER that uses 3D face shape models with depth information can capture subtle facial deformations, which are naturally robust to pose and lighting variations [10, 11]. Although some studies have tried to solve the problems caused by illumination and occlusion [12], the computer-vision-based FER still faces a huge performance challenge and constrained position problem in dealing with those variations. Therefore, more robust and position independent methods are needed so as to adapt to fairly common unconstrained scenarios.

Another more practical and privacy-secure approach is surface electromyography- (sEMG-) based FER. Facial sEMGs widely distributing on the skin [13] directly reflect the activation status of different facial action units (AUs), which can be used for inner emotion decoding according to the facial action coding system [14, 15]. Therefore, this sEMG-based FER is sensitive to subtle muscle movements and is less susceptible to environmental changes. Nevertheless, compared with the usual sEMG applications (such as gesture recognition), the sEMGs for FER are weaker and more complex with vast facial muscle involved, due to the subtleness, complexity, and variation of facial expressions. Some studies have yielded encouraging results in addressing the application of sEMG in FER. In 1984, Fridlund et al. [16] demonstrated sEMG helpful in automatic FER. Recently, a headband is specially designed to reduce the restriction of the rigid electrodes on facial expressions, but the headband can only recognize eyebrow-related facial expressions [17]. Additionally, a learning method of emotion distribution is proposed to predict the facial expression distribution more accurately [18], but the electrodes' irritation on the subject still remained unsolved. Most of the current progresses made on multichannel sEMGs [18–21] for FER still use intrinsically rigid electrodes, which pose the following challenges: firstly, the mismatch of rigid electrodes and soft skin (the deformation up to 45% [22]) makes it difficult to obtain high-fidelity sEMGs; secondly, wearing rigid electrodes on the face may hinder spontaneous facial expressions and irritates the subject. Hence, more robust sEMG acquisition methods are needed to achieve both high-fidelity signals and high wearability. With excelling mechanical and biological properties, flexible electronics have unparalleled advantages in soft, curvilinear surfaces, showing promising applications in the fields of robotic electronic skin [1, 23, 24], smart skin of aircraft [25], health care [26–28], and human-computer interaction [29–31]. Currently, most studies use flexible strain sensors for FER [28, 32]. However, when the facial muscle activity is intense but the external deformation is not obvious (e.g., clenching of teeth in anger), it will be challenging to detect valid responses by indirect strain sensors. In contrast, flexible electrodes can accurately detect the activities of facial muscles. A curve-based sensor can run complicated facial expression recognition and may contribute practical applications on auxiliary apparatus for skin micromotion manipulation for paraplegics [28]. The flexible electronics can detect the muscle activation associated with “enjoyment,” “social,” and “masked” smiles [20]. Therefore, a FER system combining soft, multichannel electrodes and an intelligent algorithm for the acquisition of facial sEMG deserves further study.

FER was originally based on machine learning for classification. In 2011, Murugappan et al. [33] presented sEMG-based human emotion classification using *K*-nearest neighbor and linear discriminant analysis. In 2018, Cai et al. [21] designed a facial expression recognition system based on sEMGs using Intel Edison board with advantages of high temporal resolution and potential flexibility of testing devices. Deep learning attempts to capture high-level abstractions through hierarchical architectures of multiple nonlinear transformations and representations and thus has made significant breakthroughs when applied for FER [34, 35]. It has also been used in expression recognition based on sEMGs. An Elman neural network that works with a specially designed headband was proposed to extract sEMG and built an emotion recognition model for recognition of facial emotional expression [17]. Recently, a novel approach based on kurtogram analysis and convolutional neural network (CNN) was proposed for the emotion classification from facial sEMGs [36]. Sensors used to collect sEMGs in FER generally have a strong sense of invasion and uncomfortable. The sensors with excellent flexibility and stretchability are becoming crucial components that can provide health monitoring systems with the capability of continuously tracking physiological signals of human body without conspicuous uncomfortableness and invasiveness [37]. The combination of flexible electrodes and intelligence algorithm provides a more portable and accurate recognition method for expression interaction in human-computer interaction.

Here, we present a robust, highly wearable FER system that obtains high-fidelity sEMGs through novel epidermal electronics that include flexible electrodes and artificial intelligence algorithm. The artificial intelligence algorithm is utilized to classify the sEMG collected by flexible electrodes. Combining deep learning algorithm and data preprocessing, 7 expressions and their 5-level intensities are accurately classified. The FER system was tested under different scenarios to meet the needs of daily use. Finally, we apply the FER system in human-avatar emotion interaction and verbal communication disambiguation, demonstrating promising prospects serving as human-computer emotional interfaces. Different from the multi-individual, position-constrained computer-vision-based FER, the proposed wearable FER system can recognize spontaneous facial expressions without the constraint of movement, space, and light, which is more suitable for the long-term mobile FER of the individual user.

## 2. Results

### 2.1. Architecture of the FER System via Epidermal Electronics

**Figure 1:**
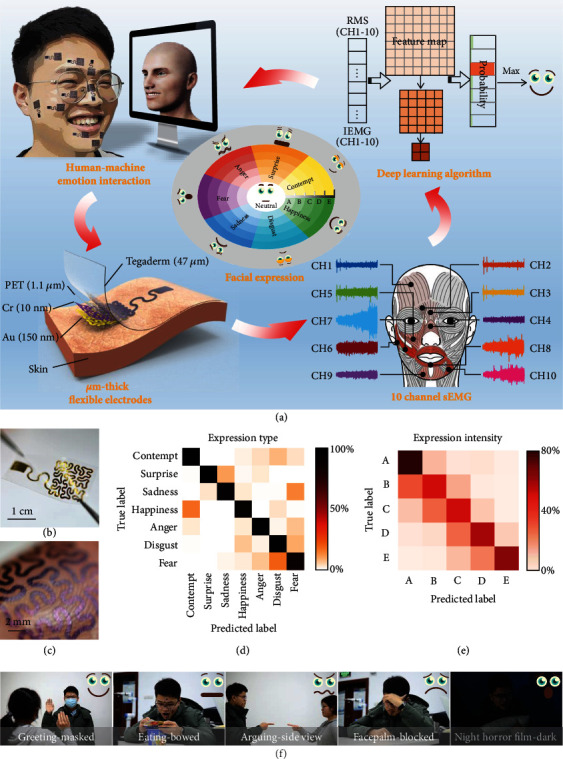
Overview of the wearable FER system. (a) Schematic illustration of human-computer expression interaction based on seven basic expressions and five levels of intensity, including the subject and avatar, *μ*m thick flexible electrodes, 10-channel sEMGs based on expression-related muscles, and the deep learning algorithm. (b) Photograph of a single flexible electrode. (c) Photograph of a flexible electrode on the fingerprint. (d, e) Confusion matrix of 7 expression types and 5 levels of expression intensities. (f) Ubiquitous applicability in various scenarios.

There are seven basic emotions in the facial action coding system, and each has its own unique and distinctive facial expressions (contempt, surprise, sadness, happiness, anger, disgust, and fear) [14]. Furthermore, the intensities of expressions are divided into 5 levels by letters A-E (from minimal to maximal intensity), and thus, we can distinguish the subjects' emotional states quantitatively, as shown in Figure 1(a). In this FER system, ten flexible electrodes are attached to the face, and the recognized expressions are transmitted to the avatar in the computer. In this way, facial expressions are taken into account in human-computer interaction, converting the cold and indifferent interaction paradigm into a warm and emotional one; for example, the avatar could respond happily when the subject is happy. The flexible electrodes are designed to be 1.2 *μ*m thick, including 150 nm thick gold (Au), 10 nm thick chromium (Cr), and 1.1 *μ*m thick polyethylene terephthalate (PET), encapsulated by a 47 *μ*m thick 3 M Tegaderm medicine tape which can be applied conformally and securely to the face with an adhesion strength of ~60 Nm^−1^. The placement of the 10 flexible electrodes is designed according to the distribution of facial muscles, where the sEMG is sensitive to facial expressions. Specifically, different facial expressions involve different muscle groups. According to the facial action coding system, 10 different action units (AU 1, AU 2, AU 4, AU 9, AU 10, AU 26, AU 12, AU 23, AU 17, and AU 15) are selected for sEMG acquisition [38], as shown in Table S1. Electrodes include 10 working electrodes, a reference electrode, and a ground electrode. The 10-channel (CH) working electrodes are attached to the corresponding AUs' positions; the reference electrode and the ground electrode are attached to the backside of the subject's two ears, respectively. Figure 1(a) shows the 10 CH sEMGs when the subject behaves happiness. As can be seen, CH6, CH8, and CH10 located at the corners of the mouth show the maximum sEMG intensity when the subject shows happiness. Because during smiling, it is mainly the AU 12 and its nearby AUs that produce contraction and drive the corners of the mouth diagonally upward. Thus, this sEMG acquisition method tailored to the muscle distribution can improve the independence between different channels. To identify the type and intensity of expressions, the features of 10 CH sEMGs are inputted into a deep learning network for training and testing. The avatar is shared with the predicted facial expression results and feedback with an appropriate emotion, such as responding to the subject's happiness by smiling, thus completing human-avatar emotional interaction.

Figure 1(b) illustrates the shape of the flexible electrodes, designed as fractal serpentine filaments to achieve the trade-off between the stretchability and the actual coverage. The flexible electrodes are soft enough to fit conformally and tightly with the curvilinear skin and its texture, as shown in Figure 1(c). The small thinness of the flexible electrodes enables excellent conformability, facilitating the acquisition of the high-fidelity facial sEMG. Thanks to the combination of ultrathin flexible electrodes and intelligent algorithms, the highest accuracy of classification in 7 expression types (Figure 1(d)) and 5 expression intensities (Figure 1(e)) reaches up to 94.48% and 79.11%, respectively. It is worth noting that most of the misidentification of intensity is due to the confusion of adjacent intensity levels, since the expression intensity level is actually a continuous process rather than a discrete one. The confusion of adjacent intensities has few impacts on the practical applications, so it is reasonable to consider the left and right cells of the diagonal of the expression intensity confusion matrix to be correctly identified. As a result, the effective accuracy of expression intensity recognition can reach 92.51%. Figure 1(f) shows some practical application scenarios, such as greeting happily, eating food with surprise, arguing angrily, face palm with sadness, and watching a night horror film in terror. Due to the masks, bowing, side view, being blocked, darkness, or other factors, the camera cannot capture a full and clear picture of the human face, so the computer-vision-based FER is difficult to apply in these complex daily situations. In contrast, our FER method still has a high recognition rate in these scenarios, as will be discussed in detail in Section 2.4.

### 2.2. Imperceptible Wearability of Epidermal Electronics

**Figure 2:**
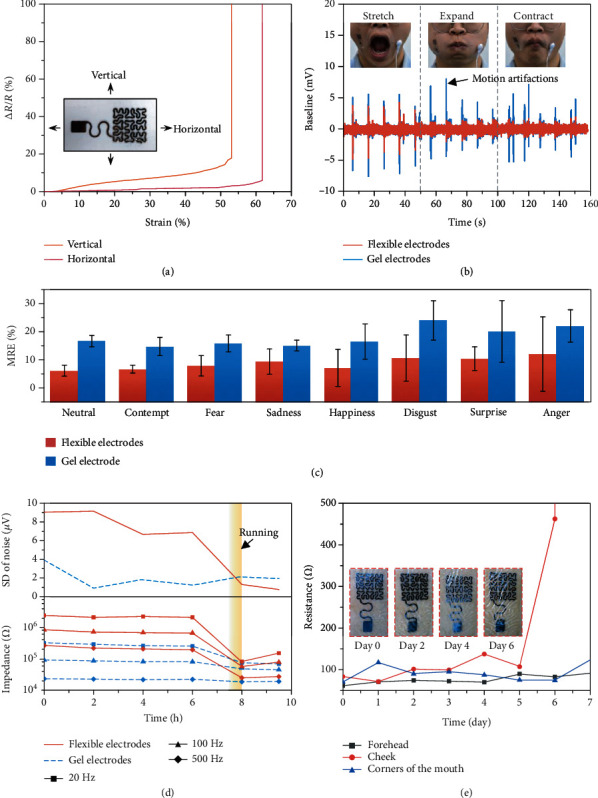
Characterization of flexible electrodes. (a) Axial stretchability of flexible electrodes. (b) Motion artifacts of flexible electrodes under various face movements after attached to the subject's face. (c) The mean relative error (MRE) of neutral and seven basic expressions before and after different electrodes were laminated. (d) Effects of prolonged wear and sweating on the electrodes' impedance and standard deviation (SD) of noise. (e) Long-term wearability and lifetime of daily use.

In order to enable intrinsically rigid Au flexibility, fractal serpentine filaments [39] and ultrathin film (1.2 *μ*m) are used to guide the design of the flexible electrodes: the former transforms tensile strain into out-of-plane deformation [40], while the latter enables the structure to be implemented more easily. Figure S1 shows the fractal serpentine filaments (0.6 mm wide) used to guide the design of the flexible electrodes. Specifically, the fractal serpentine structure is derived from Peano-based fractal geometries [41], which include 2 types of bricks: brick 1 for extending and brick 2 for making a right/left turn, respectively. The stacking of different bricks composes the stretchable fractal serpentine geometries. The coverage rate of the electrodes (the area of filament divided by the nominal overall area) can reach up to 56.18% while meeting the requirement of stretchability, which is beneficial to increasing the actual area of the working electrode on a limited overall size.  Figure 2(a) tests the tensile properties of the flexible electrodes. The serpentine-like design increases the tensile capacities of the electrodes in the *X* and *Y* directions to 62% (*X* direction) and 53% (*Y* direction), ensuring that the flexible electrodes keep working even when the tension of the human face reaches up to 45% [22]. Figure S2 is the contour of the maximum principal strain of the electrode at 45% stretch of the skin. The strains on the vast majority of the flexible electrode are less than 10% (satisfying the damage criterion for AU), and the overall strain will decrease sharply once parts of the electrode enter plasticity, which means that the flexible electrode would not be damaged despite the plastic deformation when a dramatic but transient deformation of the facial skin occurred. These soft, stretchable flexible electrodes have a more reliable contact interface with the human skin than commercial gel electrodes (2228, 3M, Germany).  Figure 2(b) illustrates the changes of sEMGs with respect to the facial deformations. The commercial gel electrodes produce noticeable motion artifacts no matter how the face is stretched, expanded, and compressed. In contrast, the motion artifacts generated by flexible electrodes are significantly reduced due to the firm contact interface [42]. Especially, the motion artifacts are basically suppressed during the expansion and compression. Due to its novel structure design, the flexible electrodes allow the acquisition of high-fidelity sEMGs for FER. In addition, we also evaluated the effect of electrode attachment on spontaneous expressions. Figure S3 is the optical photos of different expressions taken in the natural state, after the gel electrodes were attached and after the flexible electrodes were attached. It is evident that the gel electrodes have an obvious limitation on the deformation of the facial skin, resulting in a more significant difference in expression with the natural state. To further quantitatively characterize the effect of electrode attachment, facial feature points are extracted via computer vision. The restriction of expressions by electrodes is measured by the mean relative error (MRE) of the distance between facial feature points before and after electrodes attachment; a smaller MRE means less restriction of spontaneous expression.  Figure 2(c) shows the MRE after applying gel and flexible electrodes. After flexible electrodes being attached, the MRE of different facial expressions is approximately half of the gel. Therefore, these flexible electrodes do not hinder spontaneous facial expressions, which contribute to the imperceptible wearing experience.

In the long-term wearing of the flexible electrodes, it is necessary to consider the influence of sweating, scraping, and flushing. Therefore, we tested the effect of sweating on electrodes' performance during long-term wearing. Two pairs of flexible electrodes and gel electrodes were closely attached on the subject's forearm, 7 cm apart between the electrodes. The electrodes-skin impedance and background noise were recorded in nearly ten hours of wearing. The subject was demanded to run for 30 minutes to sweat at seven and a half hours.  Figure 2(d) shows that the commercial gel electrodes have lower impedance and noise during the whole test. However, the impedance and standard deviation (SD) of noise for the flexible electrodes were significantly reduced after sweating. Figure S5 shows the sEMGs generated by holding a 5 kg grip strength meter before and after running. It is evident that the signal-to-noise ratio of sEMGs acquired by the flexible electrodes is significantly improved after sweating, which means that the accumulation of sweat during long-term wearing is beneficial for the acquisition of high-fidelity signals instead. This is because sweat results in a high skin hydration level, and thus, the overall conductivity and the dielectric constant of skin are both increasing [43], which results in a reduction of the overall impedance. Therefore, the noise of sEMG is also reduced due to the lower interfacial impedance. Furthermore, applying in daily life requires excellent durability of the electrodes. Figure S6 shows the fatigue performance of the flexible electrodes, and the flexible electrodes have not been damaged after being axially extended with 18.7% applied strain (sufficient for natural motions of the skin) 10000 times. In addition, the flexible electrodes were tested on a longer time scale. The skin electrodes were attached in three specific positions of the subject (forehead, cheeks, and the corners of the mouth); the subject was demanded to take a bath every two days. The resistance and morphology were recorded every day, as shown in Figure 2(e) and Figure S7. Only the resistance of the forehead flexible electrodes was significantly increased at the sixth day (damaged at the seventh day). However, on the cheek and the corners of the mouth, there was still no significant change in electrical resistance after a week, which demonstrates that the flexible electrodes are suitable for long-term wearing.

### 2.3. sEMG-Based FER Algorithm with Deep Learning

Here, the flexible electrodes are used to acquire facial expressions' sEMGs to establish data set. The data set is based on 10 AUs (AU 1; AU 2; AU 4; AU 9; AU 10; AU 12; AU 23; AU 26; AU 15; AU 17), in which the 7 prototypical facial expressions are included. The 5 different intensities of each expression are collected. The sampling frequency of 10 channels is 1000 Hz. Data distribution of contempt under 5 intensities is shown in Figure S8. As can be seen, the signal values show a certain but not linear positive correlation with expression intensities, which means that the further algorithm is needed for accurate distinguishing. The subjects need to train their own classification model based on the first collected data. In this method, expression and intensity classifications are carried out by learning the relationship between sEMGs in 10 channels of the face.

**Figure 3:**
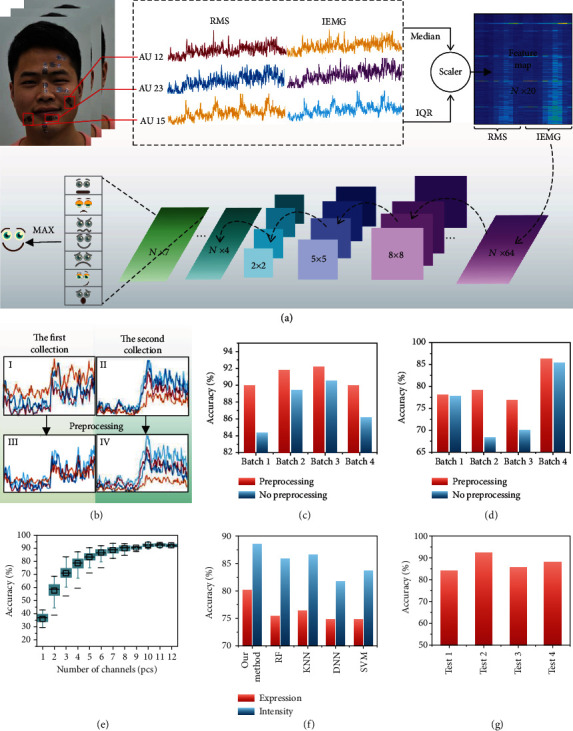
Algorithm and result comparison. (a) Schematic of FER-EE classifier. (b) The feature comparison before and after preprocessing. (c) The accuracy of expression intensity classification before and after preprocessing. (d) The accuracy of expression type classification before and after preprocessing. (e) The accuracy of FER under increasing and decreasing the number of channels. (f) The accuracy of different classification algorithms. (g) The accuracy of expression classification of four subjects.

Figure 3(a) shows the schematic of our method. We propose a wearable facial expression recognition via epidermal electronics (FER-EE) classifier, which inputs the sEMGs collected by flexible electrodes into the convolutional neural network for classification. There are three parts in the method: sEMG acquisition, preprocessing, and classification. This model is aimed at learning a mapping that translates *X*_*n*_ into an output *P*_*m*_  with formal expression as *M* : (*X*_*n*_, *T*_*n*_)⟶*P*_*m*_. sEMGs  *X*_*n*_ is defined as an input (*n* is the number of signals collected in a period) which is collected by flexible electrodes. The input is made up of signals from 10 channels which are denoted as *X*_*n*_ = (*x*_1,_ ⋯ , *x*_10_). *X*_*n*_ is scaled by the preprocessing, and thus, we augmented the input data with high intersubject variations that exist due to different personal attributes. *X*_*n*_ is transferred to *F*_*m*_ : *X*_*n*_⟶*F*_*m*_, *F*_*m*_ = (*f*_1,_ ⋯ , *f*_20_), where each *f*_*i*_ denotes a preprocessed value.  *F*_*m*_ is the input data into the convolutional neural network, where the probabilities for each category can be calculated: *P*_*m*_ = (*p*_1,_ ⋯ , *p*_*k*_)  (where *m* is the frame number with a frame frequency of 20 and *k* is the number of classification categories, *k* = 5  when the model is classified for intensity or *k* = 7 when the model is classified for expression). The time domain features and spatial domain features during the measurement period are combined to train the model. Firstly, the sEMG is analyzed in the time domain. Features in the time domain are usually extracted quickly and easily because these features do not need any transformation, which are calculated based on the time series of raw sEMG. Root mean square (RMS) and integrated electromyography (IEMG) are the target features used in our method. When a specific category of facial expression occurs, IEMG reflects the strength of different muscle activities in 10 channels, and RMS reflects the average level of muscle discharge in these channels at a particular time. RMS and IEMG can be expressed as the formulas (1) and (2) shown in Table S2, where *X*_*i*_ represents the sEMG in a segment *i* and *N* = 200 denotes the length of the sEMG. Because the signals are collected by 10 channels, there are 10 features from both RMS and IEMG. The feature matrix *F*_*m*_ = (RMS, IEMG) is composed of these 20 features.

As outliers in the sEMGs are inevitable, these abnormal values are usually regarded as the maximum or minimum values in a period, which will affect the statistical parameters such as variance and mean value of the data segment, leading to severe errors. The commonly used standardization and normalization [44] are greatly influenced by outliers, so our method takes advantage of the idea of robust scaler [45] to process features, and the procedure of feature processing is as formula (3) shown in Table S2. In formula (3), *j* ∈ [1, 20], median represents the median of *f*_*j*_, and IQR is the quartile distance of *f*_*j*_. *F*_*m*_ is obtained by removing the median and scaling the data according to the quartile range.

Figure 3(b) shows the signals before and after preprocessing for facial expression of contempt's data collected from two batches. The first row is the raw data collected from two batches, and the second row is the data after preprocessing. It can be found that there are differences between the two groups of data. Nevertheless, after preprocessing, the differences between the groups are significantly reduced. This indicates that preprocessing makes our algorithm robust to the differences between batches. Further, the effect of preprocessing on the robustness is quantitatively demonstrated. Figures 3(c) and 3(d), respectively, show the accuracy of the expression intensities and types collected from four batches before and after preprocessing by the same subject. It can be seen that each batch's accuracy has improved, the highest increased by 11.3%, and the average increased by 4.77%. In the preprocessing proposed by our method, IEMG and RMS are calculated from sEMG, and the robust scaler is used to remove the same expression's feature difference caused by the batches.

In this method, a convolution neural network is used to classify the expression and intensity of sEMGs collected based on flexible electrodes. The training model is for the individual. The data collected from the same batch was divided into two mutually exclusive sets by stratified sampling, which were, respectively, used as training set and validation set. The sample number of training set accounted for 2/3 of the total. The testing set was collected in different time periods, and thus, there was no repeated data in the data sets for training, validation, and testing. The accuracy of the model in training set, validation set, and test set is 97.22%, 92.38%, and 85.51%, respectively, as shown in Figure S9. Among them, facial expressions are classified into 7 categories, and intensity is classified into 5 categories. In FACS [14], the definitions of AU's different intensities were distinguished by words such as slightly, strong, and maximum. And in the experimental results [46] of the AU intensity estimation in computer vision method, it is found that the accuracy of intensity estimation is very uneven. Therefore, our system adopts fuzzy processing for the intensity estimation, and the recognition of adjacent intensity is also regarded as correct classification. In the test set, the average accuracy is 80.17% in expression classification and 88.58% in intensity classification. The subjects collected a sequence of facial expressions and marked the intensity of facial expressions by themselves. By comparing the recognition results with the label, the accuracy can reach 77.11%. The experimental results proved that the recognition results after fuzzy processing were in line with the subjective intensity assessment of the subjects. 10 AU channels were selected in our method. In order to better verify the robustness of the method, we discussed the accuracy of different the number of channels. The results are shown in Figure 3(e). When the number of channels decreased, the accuracy gradually decreased. However, when the number of channels was more than 8, the accuracy gradually leveled off, which means that the FER system is robust to the damage of one or two flexible electrodes. In addition, in order to validate our 10 channels for the most appropriate choice, we added two channels to collect AU6 and AU20. It was found that when the number of channels increases to 11 or 12, the accuracy does not improve. The experimental results prove that our selection of channels is efficient and streamlined. Training and testing of the models were carried out on a 3.20 GHz Core i5 PC, and the running time on the four testing sets is shown in Figure S10. Each test group had about 100 s of data, and the average predicted time of the model was 0.03817 s.

Previous studies on physiological signal classification have used SVM [47], RF [48], and other algorithms [49] for classification, while in this paper convolutional neural network is used to classify sEMGs. Figure 3(f) shows the accuracy of five classification algorithms in facial expression and intensity classification, among which convolution neural network is the algorithm with the highest accuracy. It can be proved that convolution neural network has a better performance in sEMG classification based on flexible electrodes.

Since the position of the flexible electronics cannot be absolutely the same each time and the positions of the different subject's facial features are different, the data was collected from multiple subjects for classification. sEMG recording was performed on 4 healthy volunteers (age: 22.75 ± 1.09, 3 men). All procedures for the tests in the healthy subjects were ethical, and the subjects gave informed consent. Different subjects attached with flexible electrodes are shown in Figure S11. Figure 3(g) shows the accuracy of expression classification of four random subjects, which proves that the accuracy of our method does not cause excessive deviation when the electrode is attached in different positions, all of which are above 82%. When the training set contains data from multiple subjects, the accuracy of predicting the one of them can reach 78.57%. The confusion matrixes of 4 subjects' expression type and intensity are shown in Figure S12.

### 2.4. Comparison with Computer-Vision-Based FER in Practical Scenarios

**Figure 4:**
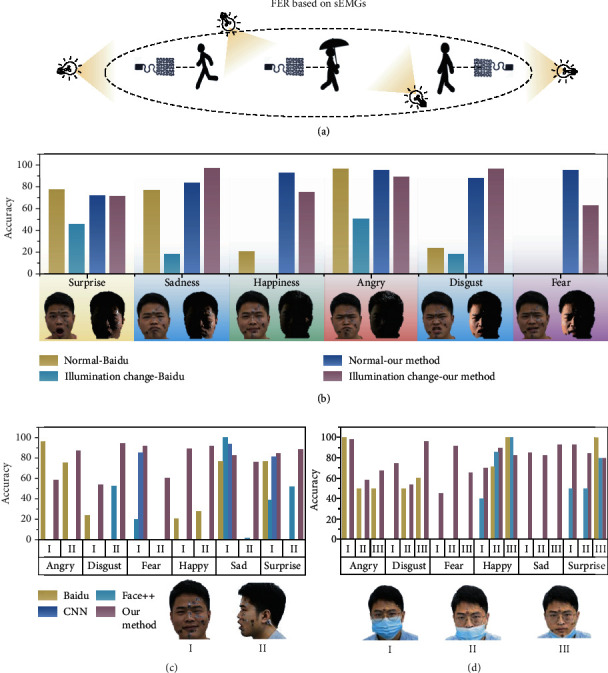
Comparison between computer vision and our method. (a) The highly wearable FER system in application scenarios. (b) The accuracy of API provided by Baidu and our method in the process of illumination change. (c) The accuracy of three methods of CV and our method under side view. (d) The accuracy of two methods of CV and our method under occlusion.

The majority of the traditional methods for FER are based on video or image, which is practical in multisubjects. These methods rely on cameras for image capture, and most of them are laboratory-controlled. The computer-vision-based methods are fragile to variations that are irrelevant to facial expressions, such as different backgrounds, illuminations, and head poses, which are fairly common in an unconstrained environment. Complementary to the computer-vision-based FER, the wearable epidermal electronics can capture subtle facial sEMGs, which is naturally robust to pose, occlusion, and lighting variations in different experimental environments, as shown in Figure 4(a). Therefore, our FER method is more suitable for individuals in mobile, long-term facial expression monitoring. Experiments conducted in this paper based on sEMGs are aimed at comparing its robustness with the state-of-the-art computer-vision-based methods. Four sets of comparative experiments were designed in this paper with various illumination, nonfrontal view, and occlusion in unconstrained environment. The sEMGs and the video were collected at the same time during the process of FER. The video is used as the test data of the computer-vision-based method, while our method takes the sEMGs as the input data. Experimental results show that epidermal electronics in this paper have good recognition accuracy in different environments, which proves that the system is highly wearable.

Figure 4(b) shows the accuracy of the expression recognition with various illumination changes. As can be seen, when the light changes gradually, the accuracy of the API provided by Baidu artificial intelligence platform [50] fluctuates greatly. The classification accuracy of computer vision and our method under low light is shown in Table S3. The reason for the misrecognition of expression in low light and dynamic light is that the facial feature information is fuzzy or even losing. Figure 4(c) shows the recognition accuracy of the four methods under side view. Compared with the normal constrained environment under side view conditions, the API provided by Baidu's artificial intelligence platform, the API provided by FACE++ [51], and the proposed network model [52] can not recognize expressions. Figure 4(d) shows the accuracy of the two methods of computer-vision-based and our method with occlusion. The subject wears masks to show 3 different areas of occlusion. As the occlusion's area increases, the accuracy of vision-based methods gradually decreases. Through a series of comparison between the computer-vision-based method and our method, the robustness and highly wearable of our method is proved under the conditions of occlusion, illumination changes, and pose variations.

### 2.5. Human-Computer Interaction Applications

**Figure 5:**
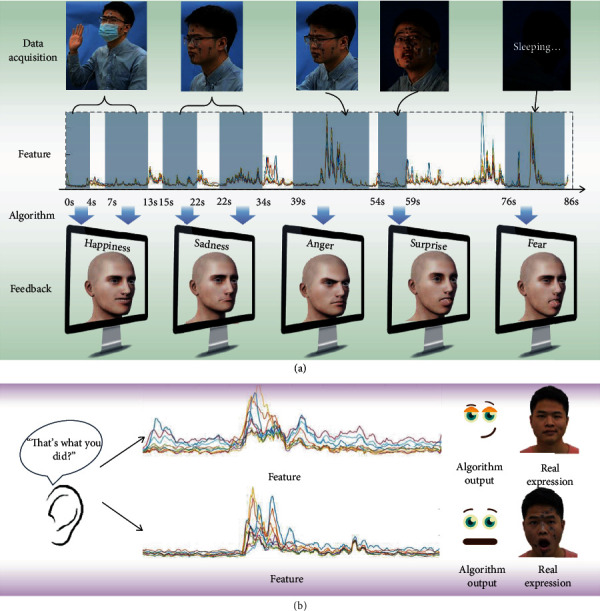
Human-computer interaction applications. (a) An example of the mobile FER system. (b) Demonstration of the auxiliary effect of our method on language emotion understanding, taking “That's what you did?” as an example which may be contempt or surprise.

Figure 5(a) demonstrates a typical application in human-avatar emotion interaction. Five different scenarios were set to imitate different situations that might be encountered in daily life, including smiling goodbye with a mask, complaining to the avatar, finding a book stolen and getting angry, the lights being turned off suddenly, and waking up from a nightmare. In this procedure, the subject's sEMG was recorded continuously by the flexible electrodes. The extracted features are shown in Figure 5(a), which are input into the FER-EE classifier for continuous expression recognition. Thus, the avatar is able to feedback with appropriate expressions to the subject by the result of FER, for example, smiling together with the subject. Movie S1 demonstrates that with our FER system, the avatar can accurately capture the subject's emotional state (such as happiness, sadness, anger, surprise, and fear) throughout the process and interact with smooth expressions; the total accuracy of which is 83.33%. The feature waveform of continuous expression is shown in Figure S13. Because sEMGs can capture the changes in muscles, the features also change significantly when the expression changes, which provides great reference for the recognition of continuous expressions. Figure S13 shows that the feature will fluctuate when the expression changes, leading to the fluctuation of the recognition result. Continuous sEMGs have outliers when the expression changes, but the recognition results are stable when the expression is stable. Excessive fluctuating data can be deleted by setting up outlier detection. This experiment proves that this highly wearable FER system can fulfill the requirements of different scenarios in daily life and is beneficial for long-term, continuous expression interaction for special users.

This FER system can not only enhance the emotional information in human-computer interaction but also enhance the understanding of natural language. When a person hears a sentence without seeing the expression of the speaker, he may misinterpret the meaning of the sentence. Take “That's what you did?” as an example. The emotion expressed in this sentence may be contempt or surprise. But when the hearing and sEMGs are combined, the emotion conveyed by the speaker can be accurately obtained, as shown in Figure 5(b). Movie S2 demonstrates the ability of this FER system to discriminate between the emotions of four different conversations, which can recognize the subject's facial expressions by collected the sEMGs via flexible electrodes. The total accuracy of eight examples in movie S2 is 85%. Our system can recognize the current expression and the real emotion of the speaker which is hoped to enhance the understanding of the interaction process. It is proved that this system is expected to assist the speech recognition system to monitor the emotion of the subjects from facial expression and language.

## 3. Discussion

We proposed a novel FER strategy designing by highly wearable deep-learning-assisted, soft epidermal electronics, which is robust for various scenarios. Based on epidermal electronics with intelligent algorithm and flexible electrodes, the FER system achieved accurate recognition on 7 expressions and 5 levels of intensity by capturing facial movements. The flexible electrodes do not hinder spontaneous expressions and can be worn for a week-long time. Since AUs are controlled by facial muscles, the electrodes are designed to capture subtle muscle movement corresponding to the specific AUs which are crucial to emotion understanding. To our knowledge, it is the first time to use epidermal electronics with AUs for FER. The proposed system is based on ground-truth AU to obtain AU measurement, and it avoids accurate AU annotation which requires expertise and time. The novelty of capturing AUs and intensity will enhance facial expression data for database construction. The combination of data preprocessing and deep learning suppresses differences of batches and individuals. The validation sets in the subject-dependent models due to the limited amount of data. Therefore, if the amount of training data is expanded, the accuracy will be improved. High-accuracy FER was accomplished in different scenarios such as illumination changing, side view, and occlusion.

Computer-vision-based FERs can recognize facial expressions of multiple people using only one camera. However, it has high requirements on the posture of the subjects and the surrounding environment and additionally shows close restraint on movement of a person. By comparison, the wearable FER system proposed can recognize continuous facial expression of a specific person for a long time and is robust to the surrounding environment and posture, which is expected to be complementary to the computer vision in the field of FER. Experiments on human-avatar emotion interaction and language disambiguation were carried out, demonstrating the application prospect of the FER system in human-computer interaction and aiding verbal communication. Nevertheless, the development and integration of wireless communication components are under further study to address the problem of Bluetooth data transmission due to the large quantity of flexible electrodes. As a result, this paper focuses on the proof-of-concept demonstration of the novel wearable FER system using a wired method. Further research may focus on a system-level integration, and sEMG can also be used as a complement to visual or verbal signals, combining their respective features and advantages with being of more excellent value in multimodal human-computer interaction.

## 4. Method

### 4.1. Fabrication of the Flexible Electrodes

A 1.1 *μ*m thick PET film (Nanyang technology, China) was laminated on the wetted tattoo paper (Huizhou Yibite Technology, China). The tattoo paper was dried at 65°C for 1 hour and further dried at 105°C for 2 hours, followed by thermal evaporation of 10 nm Cr and 150 nm Au. Then, the film was patterned by a programmable mechanical cutter (CE6000-40, GRAPHTEC, Japan). The patterned electrodes are transferred to the thermally released tape (TRT) (REVALPHA, Nitto, Japan) by spraying water on the back of the tattoo paper. The TRT is deactivated at 130°C for ~3 min. Finally, the flexible electrodes are transferred from the deactivated TRT to 3 M Tegaderm by Rolling the TRT from one side to the other. This transfer method can effectively prevent the TRT and Tegaderm from sticking. More importantly, this pure rolling transfer method also minimizes the strain [13], which benefits the yield of flexible electrode manufacturing.

### 4.2. MRE Calculation of Facial Feature Points

As shown in Figure S3, the picture of neutral state and seven basic facial expressions was taken (5 independent pictures for each expression). Then, the computer vision method was used to extract the facial feature points (as shown in Figure S4, a total of 38 points). The feature point of the nasal tip is set as the base point. The distances *l*_*i*_ from the other 37 feature points to the base point represent the geometric features of the face. Δ*l*_*i*_ is the corresponding difference of *l*_*i*_ in each feature point after electrodes are attached. The mean relative error (MRE) of Δ*l*_*i*_ is used to quantify the restriction of the electrodes to the facial expressions.

### 4.3. The Structure of FER-EE Classifier

In the process of classification, the model is trained by combining the features *F*_*m*_ from 10 channels in the spatial domain. The FER-EE classifier contains full connection layers, convolution layer, and pooling layer. The preprocessed features *F*_*m*_ is the input data of the network. This process maps 20 feature vectors *v*_1,64_ into a feature map *m*_8×8_ for classification. The full connection layer is responsible for getting a *n* × 64 matrix. The 1 × 64 vector is remapped to an 8 × 8 matrix and then taken into the convolution layer using a 2 × 2 filter and becomes a 5 × 5 matrix. The maximum pooling layer takes a 4 × 4 filter for downsampling. The matrix is finally put into the flatten layer and thus get a 1 × 64 vector. Finally, softmax function is used by a full connection layer as the output layer. Softmax function is often used as activation function in the last layer of multiclassification algorithm. The last layer computes the lost and outputs the probabilities of all expressions in the current prediction. *P* = (*p*_1_, ⋯, *p*_*k*_) is the output of the function, where *p*_*j*_ is the probability value of *v* representing the *j*th expression and *v* is the softmax function's input. Each layer is trained by backpropagation. The cross-entropy loss function is used to calculate the loss between the predicting value and true value. Finally, we calculate the maximum value of *P*: MAX_*j*=1_^*k*^(*P*_*j*_)⟶*j*, *j*  can be mapped to the corresponding expression.

## Data Availability

All data are available in the manuscript or supplementary materials or from the author.

## Supplementary Materials

Supplementary 1 Figure S1: the flexible electrode on Tegaderm and the design of the fractal serpentine structure. Figure S2: the strain distributions of flexible electrodes under horizontal (left) and vertical (right) tensing with 45% by FEA. Figure S3: the facial expressions and deformations of electrodes under normal state, with flexible electrodes laminated and with gel electrodes laminated. Figure S4: the 38 different facial feature points exacted by computer vision. Figure S5: the sEMG of holding a 5 kg grip strength meter before and after running. Figure S6: resistance change under 18.60% axial cyclic stretching. Figure S7: the flexible electrodes' morphology changes in long-term wearing, taking a shower every two days. Figure S8: data distribution of contempt under 5 intensities. Figure S9: the accuracy of our method in data sets for training, validation, and testing. Figure S10: the running time of the classification algorithm. Figure S11: different subjects attached flexible electrodes.Figure S12: confusion matrixes of 4 subjects' expression type and intensity. Figure S13: features of sEMGs and recognition results in a segment of continuous expression transformation. Table S1: correspondence of sEMG channels, AUs, muscular basis, and related emotions. Table S2: the time domain and frequency domain characteristic formulas are used. Table S3: the classification accuracy of computer vision and our method under low light.

Supplementary 2 Movie S1: human-avatar emotion interaction in different scenarios of daily life.

Supplementary 3 Movie S2: four typical conversations demonstrating the application in verbal communication disambiguation.
