# Supplementary material for: Highly Robust and Wearable Facial Expression Recognition via Deep-Learning-Assisted, Soft Epidermal Electronics
Source: Research (Wash D C). 2021 Jul 15;2021:9759601. doi: 10.34133/2021/9759601 (PMC8302843; doi:10.34133/2021/9759601)
Supplement: Supplementary 1 — Figure S1: the flexible electrode on Tegaderm and the design of the fractal serpentine structure. Figure S2: the strain distributions of flexible electrodes under horizontal (left) and vertical (right) tensing with 45% by FEA. Figure S3: the facial expressions and deformations of electrodes under normal state, with flexible electrodes laminated and with gel electrodes laminated. Figure S4: the 38 different facial feature points exacted by computer vision. Figure S5: the sEMG of holding a 5 kg grip strength meter before and after running. Figure S6: resistance change under 18.60% axial cyclic stretching. Figure S7: the flexible electrodes' morphology changes in long-term wearing, taking a shower every two days. Figure S8: data distribution of contempt under 5 intensities. Figure S9: the accuracy of our method in data sets for training, validation, and testing. Figure S10: the running time of the classification algorithm. Figure S11: different subjects attached flexible electrodes.Figure S12: confusion matrixes of 4 subjects' expression type and intensity. Figure S13: features of sEMGs and recognition results in a segment of continuous expression transformation. Table S1: correspondence of sEMG channels, AUs, muscular basis, and related emotions. Table S2: the time domain and frequency domain characteristic formulas are used. Table S3: the classification accuracy of computer vision and our method under low light. [file 9759601.f1.docx]

Supplementary Materials

**Highly Robust and Wearable Facial Expression Recognition via Deep-Learning-Asissted, Soft Epidermal Electronics**

Meiqi Zhuang^1^†, Lang Yin^2,3^†, Youhua Wang^2,3^, Yunzhao Bai^2,3^, Jian Zhan^2,3^, Chao Hou^2,3^, Liting Yin^2,3^, Zhangyu Xu^2,3^, Xiaohui Tan^1^*, YongAn Huang^2,3^*

^1^ Information Engineering College, Capital Normal University, Beijing 100048, China.

^2^ State Key Laboratory of Digital Manufacturing Equipment and Technology, Huazhong University of Science and Technology, Wuhan 430074, China.

^3^ Flexible Electronics Research Center, Huazhong University of Science and Technology, Wuhan 430074, China.

† These authors contributed equally to this work

* Correspondence should be addressed to Xiaohui Tan; xiaohuitan@cnu.edu.cn and YongAn Huang; [yahuang@hust.edu.cn](mailto:yahuang@hust.edu.cn)


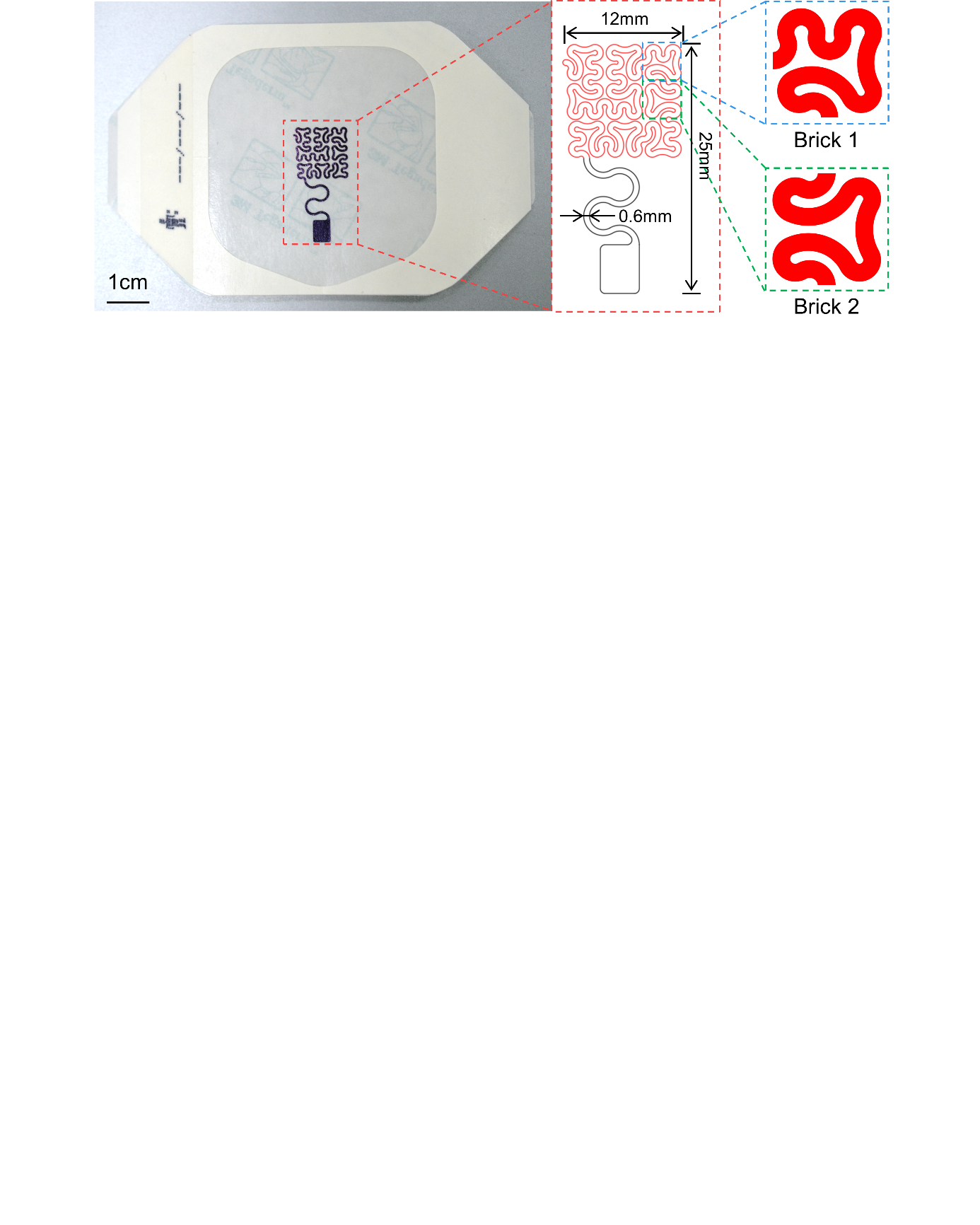


***Figure S1. The flexible electrode on Tegaderm, and the design of the fractal serpentine structure.***

***
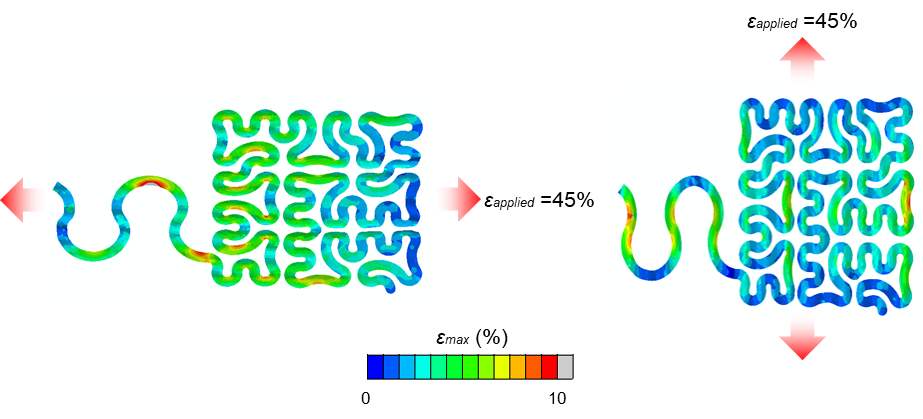
***

***Figure S2. The strain distributions of flexible electrodes under horizontal (left) and vertical (right) tensing with 45% by FEA.***


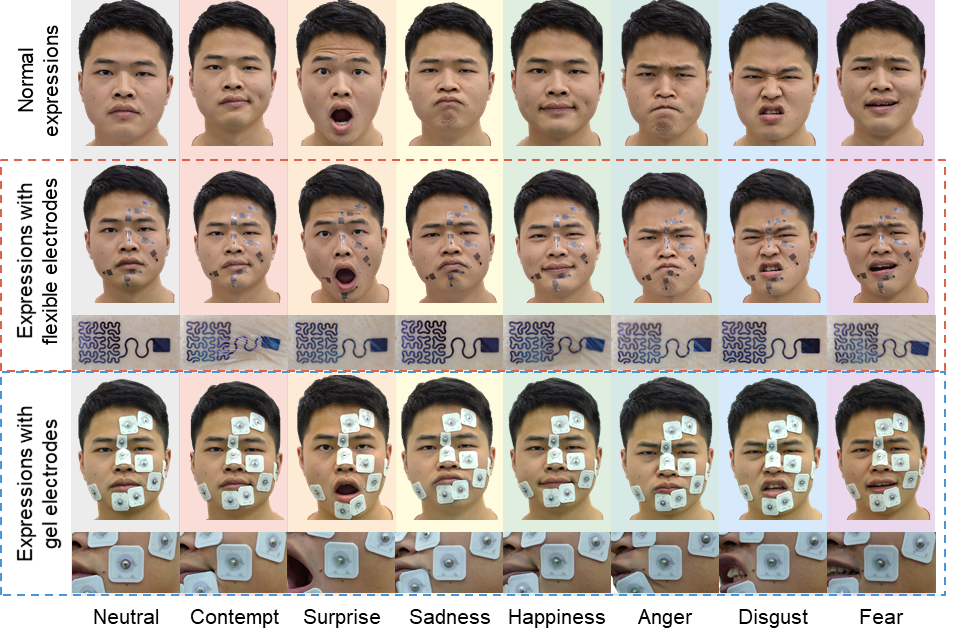


***Figure S3. The facial expressions and deformations of electrodes under normal state, with flexible electrodes laminated and with gel electrodes laminated.***

***
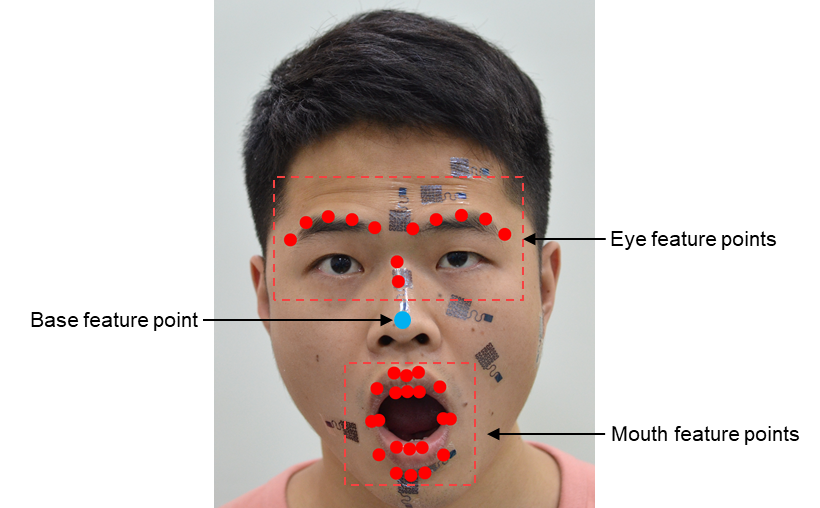
***

***Figure S4. The 38 different facial feature points exacted by computer vision.
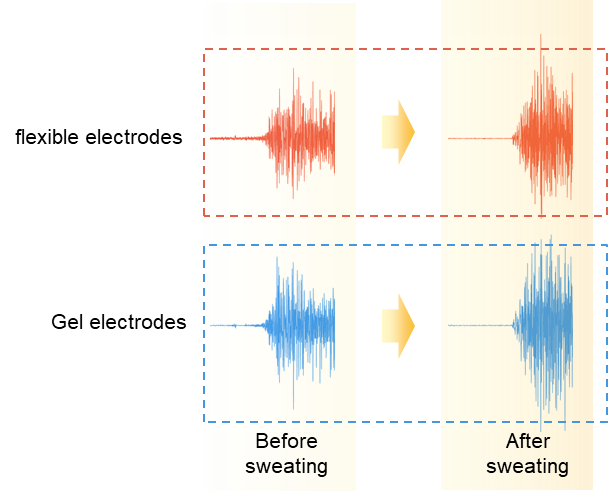
***

***Figure S5. The sEMG of holding a 5kg grip strength meter before and after running.***

***
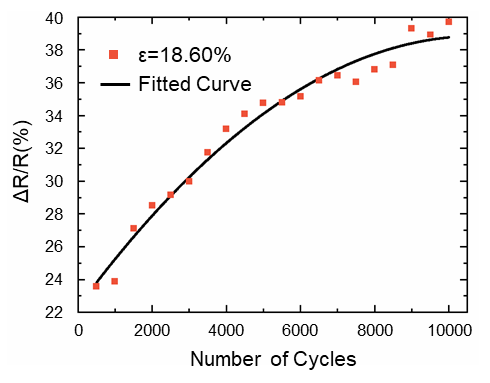
***

***Figure S6. Resistance change under 18.60% axial cyclic stretching.***

***
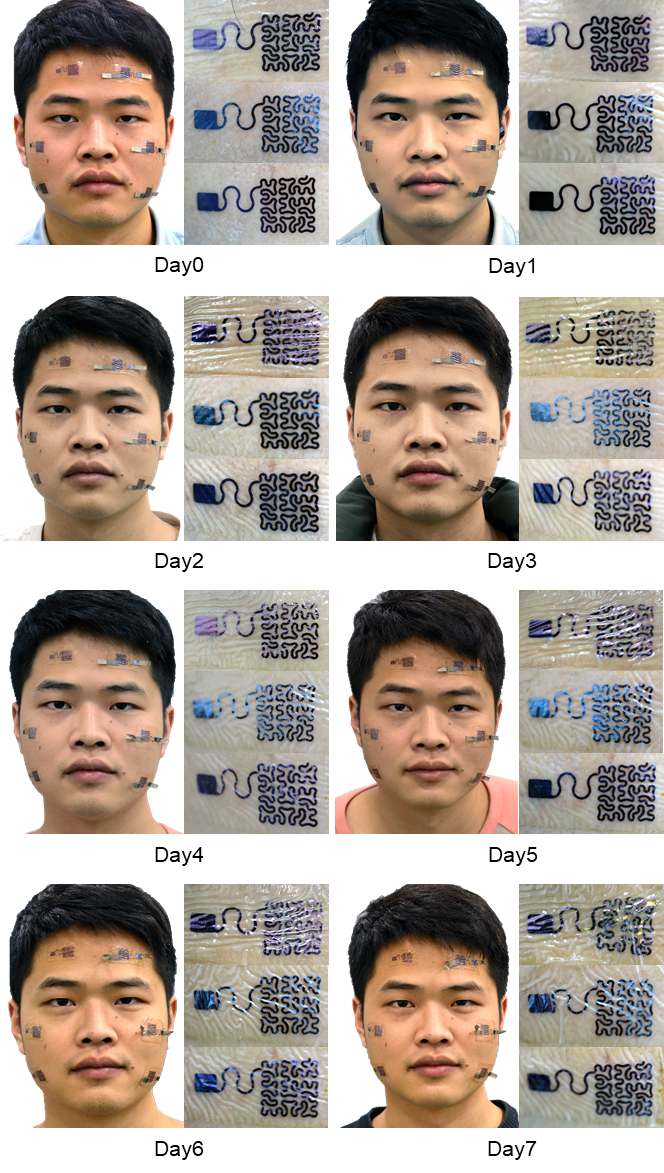
***

***Figure S7. The flexible electrodes’ morphology changes in long-term wearing, taking a shower every two days.***


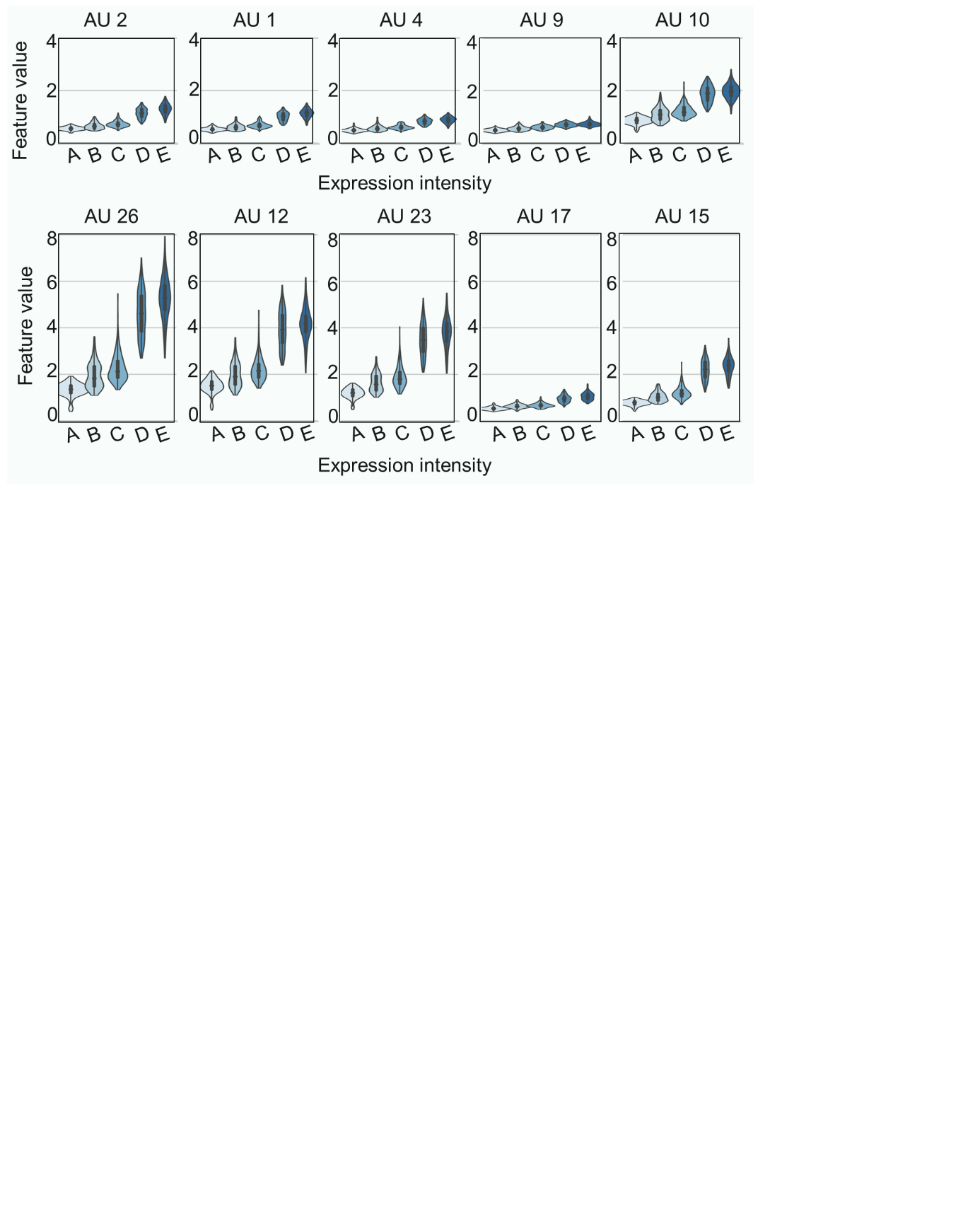


***Figure S8. Data distribution of contempt under 5 intensities.***


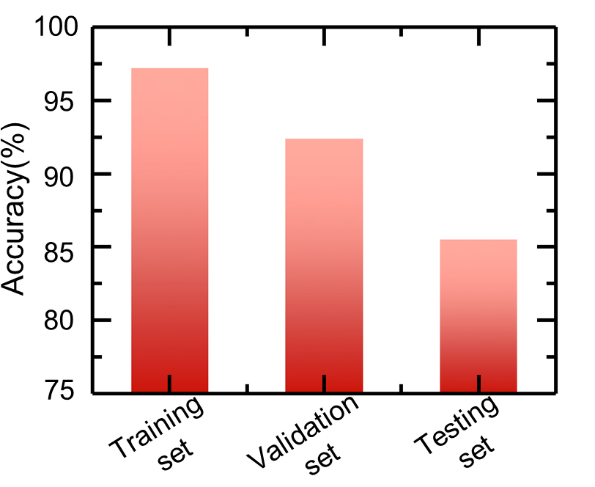


***Figure S9.*** ***The accuracy of our method in data sets for training, validation, and testing.***


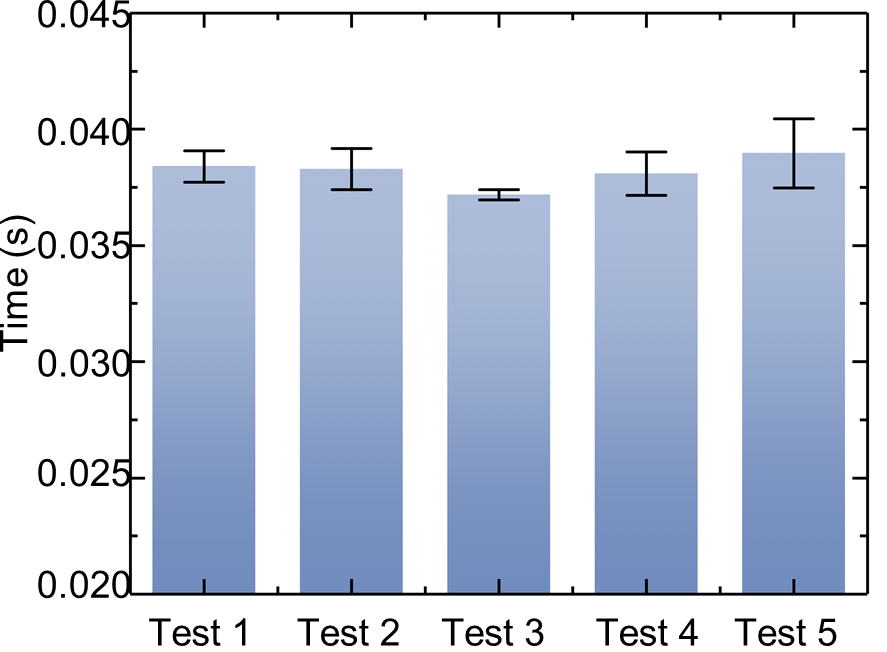


***Figure S10. The*** ***running time of the classification algorithm.***


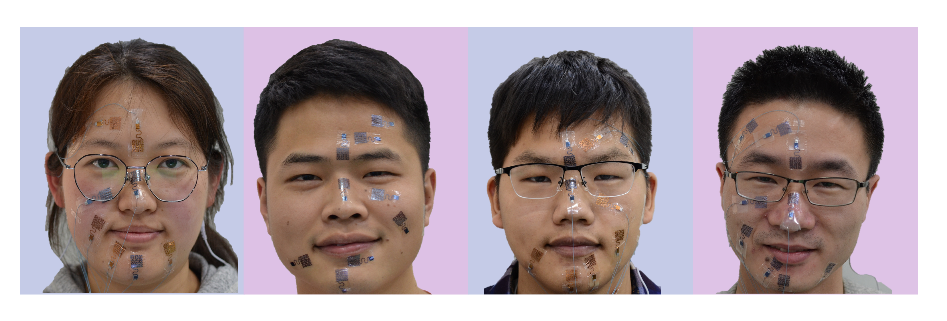


***Figure S11. Different subjects attached flexible electrodes.***


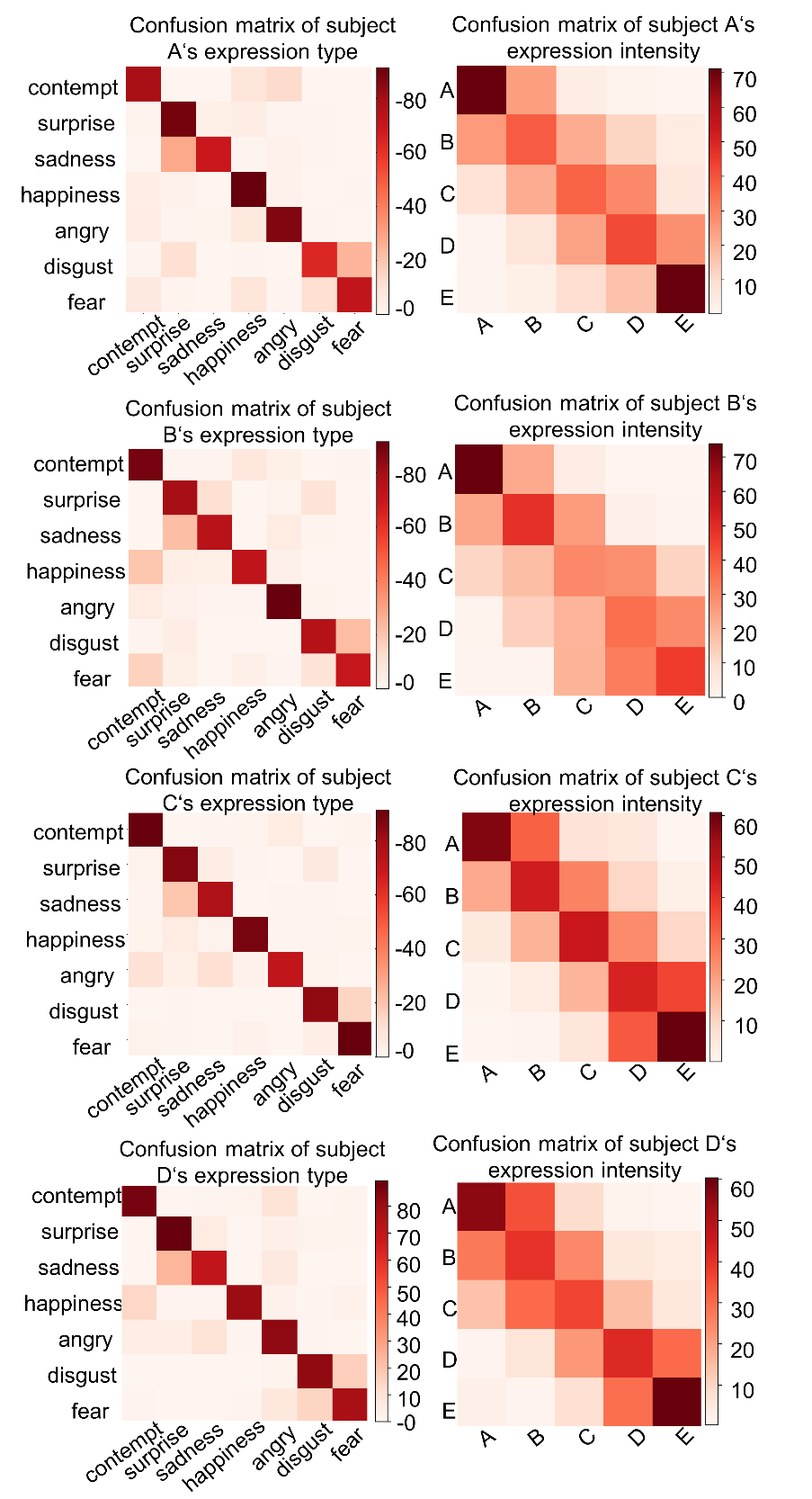


***Figure S12. Confusion matrixes of 4 subjects’ expression type and intensity.***


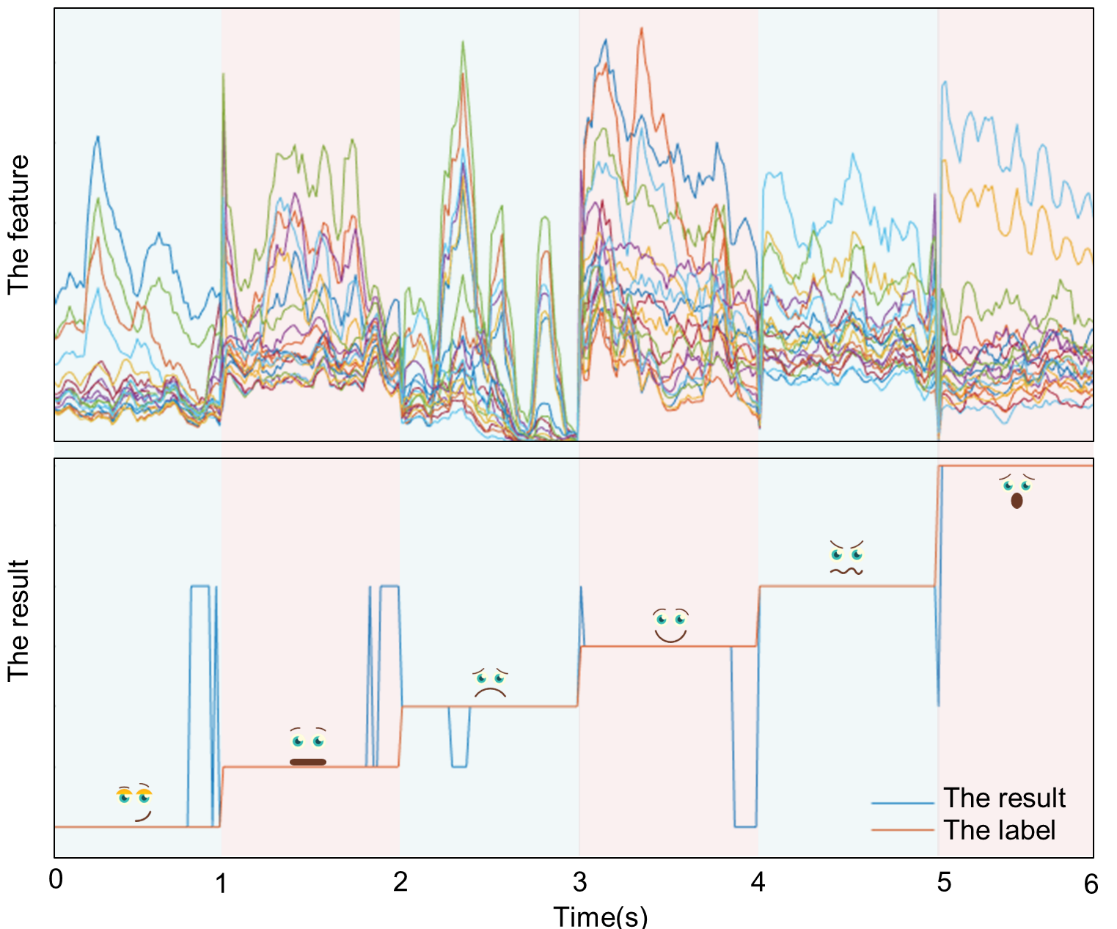


***Figure S13. Features of sEMGs and recognition results in a segment of continuous expression transformation.***

***Table S1. Correspondence of sEMG channels, AUs, Muscular basis, and related emotions****.*

| **Channels** | **Action Units** | **Muscular basis** | **Related emotions** |
| --- | --- | --- | --- |
| **Ch1** | AU 1 | Frontal muscle | Sadness, Surprise, Fear |
| **Ch2** | AU 2 | Frontal muscle | Surprise, Fear |
| **Ch3** | AU 4 | Coiter muscle | Sadness, Fear, Anger |
| **Ch4** | AU 9 | Nasalis | Disgust |
| **Ch5** | AU 10 | Levator labii superioris | Disgust |
| **Ch6** | AU 26 | Musculus masseter | Surprise, Fear |
| **Ch7** | AU 12 | Buccinator | Happiness, Contempt |
| **Ch8** | AU 23 | Orbicular muscle of mouth | Anger |
| **Ch9** | AU 17 | Depressor anguli oris | Sadness, Disgust |
| **Ch10** | AU 15 | Mentalis | Sadness, Disgust |

***Table S2. The time domain and frequency domain characteristic formulas are used.****.*

| **Number** | **Formula** |
| --- | --- |
| **(1)** | $RMS=\sqrt{\frac{1}{N}\sum_{i=1}^{N} X_{i}^{2}}$ |
| **(2)** | $IEMG=\sum_{i=1}^{N} \left\vert X_{i} \right\vert$ |
| **(3)** | $\left\{ \begin{aligned} f_{j}^{'}=\frac{f_{j}-median}{IQR} \\ F_{m}=\left( f_{1}^{'},\ldots,f_{20}^{'} \right) \end{aligned} \right.$ |

***Table S3. The classification accuracy of computer vision and our method under low light.***

|  | **Normal-Baidu** | **Low light-Baidu** | **Normal-FACE++** | **Low light-FACE++** | **Normal-CNN** | **Low light-CNN** | **Normal-our method** | **Our method** |
| --- | --- | --- | --- | --- | --- | --- | --- | --- |
| **Anger** | 96.34 | 45.35 | 0 | 14.63 | 0 | 29.27 | 87.05 | 88.69 |
| **Disgust** | 23.46 | 0 | 0 | 17.28 | 0 | 0 | 94.48 | 95.95 |
| **Fear** | 0 | 0 | 19.75 | 0 | 85.19 | 36.14 | 60.61 | 63.09 |
| **Happiness** | 20.48 | 2.47 | 0 | 0 | 0 | 0 | 91.31 | 75.13 |
| **Sadness** | 76.54 | 72.84 | 100 | 97.5 | 93.83 | 97.5 | 75.87 | 96.8 |
| **Surprise** | 76.83 | 20 | 39.02 | 53.75 | 80.72 | 98.77 | 88.42 | 71.39 |
